# Pharmacologic Tumor PDL1 Depletion with Cefepime or Ceftazidime Promotes DNA Damage and Sensitivity to DNA-Damaging Agents

**DOI:** 10.3390/ijms23095129

**Published:** 2022-05-04

**Authors:** Clare Murray, Eva Galvan, Carlos Ontiveros, Yilun Deng, Haiyan Bai, Alvaro Souto Padron, Kathryn Hinchee-Rodriguez, Myrna G. Garcia, Anand Kornepati, Jose Conejo-Garcia, Tyler J. Curiel

**Affiliations:** 1 Graduate School of Biomedical Science, University of Texas Health, San Antonio, TX 78229, USA; murrayc1@livemail.uthscsa.edu (C.M.); ontiverosc@livemail.uthscsa.edu (C.O.); garciam64@livemail.uthscsa.edu (M.G.G.); kornepati@livemail.uthscsa.edu (A.K.); 2 UT Health Mays Cancer Center, University of Texas Health, San Antonio, TX 78229, USA; galvane@uthscsa.edu; 3 Department of Radiation Oncology, University of Texas Health, San Antonio, TX 78229, USA; 4 Department of Medicine, University of Texas Health, San Antonio, TX 78229, USA; dengy@uthscsa.edu (Y.D.); baih@uthscsa.edu (H.B.); soutopadron@uthscsa.edu (A.S.P.); khinchee@gmail.com (K.H.-R.); 5 Department of Immunology, Moffitt Cancer Institute, Tampa, FL 33612, USA; jose31.conejo-garcia@moffitt.org

**Keywords:** PDL1, immunotherapy, DNA damage, drug repurposing, β-lactam antibiotics

## Abstract

The interaction between tumor surface-expressed PDL1 and immune cell PD1 for the evasion of antitumor immunity is well established and is targeted by FDA-approved anti-PDL1 and anti-PD1 antibodies. Nonetheless, recent studies highlight the immunopathogenicity of tumor-intrinsic PDL1 signals that can contribute to the resistance to targeted small molecules, cytotoxic chemotherapy, and αPD1 immunotherapy. As genetic PDL1 depletion is not currently clinically tractable, we screened FDA-approved drugs to identify those that significantly deplete tumor PDL1. Among the candidates, we identified the β-lactam cephalosporin antibiotic cefepime as a tumor PDL1-depleting drug (PDD) that increases tumor DNA damage and sensitivity to DNA-damaging agents *in vitro* in distinct aggressive mouse and human cancer lines, including glioblastoma multiforme, ovarian cancer, bladder cancer, and melanoma. Cefepime reduced tumor PDL1 post-translationally through ubiquitination, improved DNA-damaging-agent treatment efficacy *in vivo* in immune-deficient and -proficient mice, activated immunogenic tumor STING signals, and phenocopied specific genetic PDL1 depletion effects. The β-lactam ring and its antibiotic properties did not appear contributory to PDL1 depletion or to these treatment effects, and the related cephalosporin ceftazidime produced similar effects. Our findings highlight the rapidly translated potential for PDDs to inhibit tumor-intrinsic PDL1 signals and improve DNA-damaging agents and immunotherapy efficacy.

## 1. Introduction

Programmed death ligand 1 (PDL1, CD274) is a B7 homology family immune cosignaling molecule that interacts with T cell PD1 to blunt antitumor immunity [1,2]. Antibodies that block the PDL1, PD1, or CTLA4 immune checkpoints are collectively termed “immune checkpoint blockade” (ICB), which is highly effective as cancer immunotherapy, but only in select patients [2,3,4,5]. ICB against cell-surface-expressed PDL1 is well known and investigated, but recent evidence for immunopathogenic tumor-intrinsic PDL1 signals demonstrates additional PDL1 effects that are important in treatment resistance and that are aside from its canonical surface role [6]. Genetic tumor PDL1 depletion suppresses pathologic tumor-intrinsic PDL1 signals and can improve the efficacy of cytotoxic agents and small molecules in distinct cancers [7,8,9,10], and genetic PDL1 cytoplasmic tail deletion in tumor cells heightens αPD1 ICB efficacy [11,12]. We reported that tumor PDL1 can also promote stemness and mTORC1 signals and suppress autophagy [7,8,13], all of which are clinically actionable targets. However, it is not currently possible to genetically deplete tumor PDL1 in cancer patients as cancer treatment.

We recently reported that genetic tumor PDL1 depletion reduced homologous recombination DNA damage repair, increased DNA damage, and improved tumor PARP inhibitor (PARPi) and gemcitabine sensitivity [14], which expands the current knowledge of tumor-intrinsic PDL1 functions in treatment resistance. αPDL1 was unable to sensitize tumors to PARPi [14], which highlights the need to investigate other methods to deplete tumor PDL1 for clinical applications. As genetic PDL1 depletion is not currently clinically actionable, we hypothesized that pharmacologic tumor PDL1 depletion could inhibit tumor-intrinsic PDL1 signals to replicate genetic PDL1 depletion effects on DNA damage and sensitize tumors to DNA-damaging agents. To identify pharmacologic tumor PDL1-depleting drugs (PDDs), we screened the Prestwick and LOPAC molecule libraries, which are enriched for FDA-approved drugs, in order to identify those that depleted tumor PDL1 in distinct mouse and human cancer cell lines. We validated the fourth-generation β-lactam cephalosporin antibiotic cefepime, which is FDA-approved to treat serious Gram-negative bacterial infections [15], as a *bona fide* PDD. We chose to study cefepime in further detail as it is inexpensive, relatively safe, and many FDA-approved β-lactams are available for the initial structure–activity-relationship studies.

We investigated whether cefepime could improve the standard-of-care DNA-damaging agents, or those currently in clinical trial, in aggressive and treatment-resistant tumor types, including glioblastoma multiforme (GBM), melanoma, ovarian cancer, and bladder cancer. Gemcitabine is a standard-of-care cytotoxic chemotherapy for bladder cancer treatment, and temozolomide (TMZ) is a standard-of-care cytotoxic chemotherapy agent for GBM [16,17]. Chk1 inhibitors (Chk1i) enhance the cytotoxic-drug and/or radiation-therapy efficacy by inhibiting cell cycle checkpoints and DNA-damage repair, and they are currently in cancer clinical trials [18]. We hypothesized that the combination of cefepime, which replicates the genetic PDL1-depletion effects on DNA damage, with other DNA-damaging agents could improve cancer treatment response.

Here, we show that cefepime phenocopies genetic PDL1 depletion in robustly increasing DNA damage and increasing sensitivity to distinct cytotoxic drugs and small molecule DNA-damage-inducing Chk1 inhibitors in various aggressive cancer cell lines, including glioblastoma multiforme, melanoma, bladder cancer, and ovarian cancer. Cefepime induced tumor STING signals, which could improve the ICB efficacy, and it treated distinct cancers *in vivo* in both an immune-independent manner and in wild-type mice, where it elicited distinct immune effects. The structurally related FDA-approved β-lactam antibiotic ceftazidime was also validated as a PDD that improved DNA-damaging agent efficacy. As cefepime is FDA-approved and generally well tolerated at doses that are needed to augment the DNA-damaging agent efficacy, it could be rapidly advanced clinically. Here, we provide the proof-of-concept that pharmacologic tumor PDL1 depletion can overcome treatment resistance to selected agents, which merits much additional study. Additional PDDs also merit further study to define the best agents for initial human clinical translation.

## 2. Results

### 2.1. Cefepime Is a Pharmacologic Tumor PDL1-Depleting Drug

**Figure 1:**
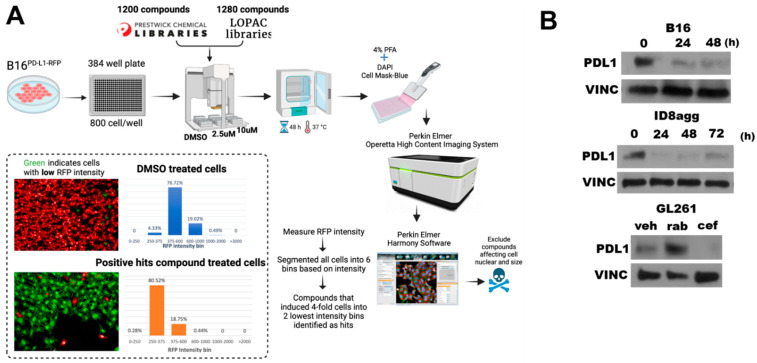
**Cefepime depletes tumor PDL1 in multiple distinct tumor cell lines.** (**A**) Schematic of high-throughput drug screen used to identify pharmacologic PDL1-depleting agents (PDDs). (**B**) Immunoblots of PDL1 and the loading control vinculin (VINC) in B16, ID8agg, and GL261 cells treated with 80 μM cefepime for indicated times. GL261 cells were treated with DMSO (veh), 250 nM rabusertib (rab), or 80 μM cefepime (cef) for 48 h. Cefepime was replenished daily.

To identify FDA-approved drugs that deplete tumor PDL1, we undertook high-throughput drug screening of molecules in the Prestwick and LOPAC libraries, which are enriched for FDA-approved agents (Figure 1A). Briefly, we treated mouse B16 melanoma cells expressing RFP-tagged PDL1 with candidate agents, and we assessed the loss of the RFP signal but not cell viability. A total of 15 candidate drugs from the screen were validated as PDL1-depleting drugs (PDDs) by flow cytometry for PDL1 expression reduction. Among the top hits was cefepime, which potently reduced the tumor PDL1 from RFP-B16 cells by flow cytometry. To validate the flow cytometry data, we used immunoblots of the total PDL1 content in various tumor lines (Figure 1B), which confirmed cefepime as a *bone fide* tumor PDD in all of the cell lines tested. Easily used murine transplantable or human tumor lines that represent difficult-to-treat human cancers and express PDL1 were selected for detailed studies. We selected cefepime among the identified PDDs on the basis of its relative efficacy in tumor PDL1 depletion, its FDA approval, its relative safety, its low cost, and the existence of many commercially available β-lactam molecules for the initial structure–activity-relationship studies.

### 2.2. Cefepime Induces DNA Damage and Sensitizes to DNA-Damaging Agents In Vitro

**Figure 2:**
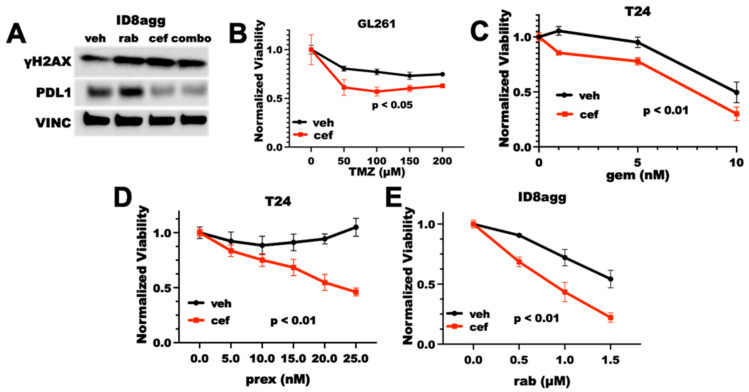
**Cefepime induces DNA damage and sensitizes to DNA-damaging agents.** (**A**) Immunoblot for γH2AX, PDL1, and vinculin loading control (VINC) of ID8agg cells treated with DMSO vehicle (veh), rabusertib (rab) as indicated, 80 μM cefepime (cef), or combination (combo) for 72 h. (**B**) MTT viability assay of GL261 cells treated with 100 μM cefepime (cef) or vehicle (DMSO) in combination with indicated concentrations of temozolamide (TMZ). *p* value by two-way ANOVA. (**C**) MTT viability of T24 cells treated with 80 μM cefepime or vehicle (DMSO) in combination with indicated concentrations of gemcitabine (gem). *p* value by two-way ANOVA. (**D**) MTT viability of T24 cells treated with the Chk1i prexasertib (prex) at indicated concentrations combined with DMSO vehicle or 80 μM cefepime. *p* value by two-way ANOVA. (**E**) MTT viability of ID8agg cells treated with rabusertib (rab) at indicated concentrations in combination with DMSO or 80 μM cefepime. *p* value by two-way ANOVA. Drugs were not replenished in these assays.

We and others have reported that tumor cells overexpress PDL1 in response to DNA damage, and we have shown that genetic tumor PDL1 depletion increases DNA damage and sensitizes tumors to DNA-damaging agents [14,19]. Thus, we investigated whether cefepime could overcome tumor PDL1 induction after DNA damage and whether it could sensitize resistant tumor cells to DNA damage-inducing drugs. We examined standard-of-care treatments, such as temozolomide for glioblastoma multiforme (GBM) and gemcitabine for bladder cancer, as well as more recently developed Chk1 inhibitors (Chk1i). Chk1i are currently in clinical trial as monotherapy and in combination with DNA-damaging chemotherapy or radiotherapy with preclinical success [18]. We reported that DNA-damaging agents increase PDL1 various tumor cell lines [14] and here in the mouse GL261 GBM line using rabusertib (Figure 1B), demonstrating that these cell lines do not have an atypical PDL1 response to DNA damage. Cefepime significantly depleted the PDL1 in ID8agg murine ovarian cancer cells, and it mitigated the PDL1 overexpression in response to DNA damage caused by the Chk1i rabusertib (Figure 2A), which suggests that PDDs could overcome PDL1 overexpression as a result of DNA damage. Furthermore, cefepime as a single agent induced γH2AX, which is a marker for DNA damage, similar to the DNA-damaging agent rabusertib (Figure 2A). On the basis of these data, we anticipated that, as the cefepime depleted tumor PDL1, it would induce DNA damage and further sensitize tumors to DNA-damaging agents. Consistent with our predictions, cefepime improved temozolomide efficacy in GL261 (Figure 2B) and gemcitabine in T24 human bladder cancer cells (Figure 2C), but modestly. By contrast, cefepime strikingly improved the efficacy of the Chk1i prexasertib in T24 cells and the Chk1i rabusertib in ID8agg cells (Figure 2D,E). However, cefepime did not sensitize MB49 mouse bladder cancer cells to PARPi (Supplementary Figure S1). Thus, cefepime induces DNA damage and sensitizes resistant mouse and human tumor cells to distinct DNA-damaging agents in tumor lines from differing cancers.

### 2.3. Cefepime-Induced DNA Damage and Synthetic Lethality Is Tumor-Cell-PDL1-Dependent and Can Include ROS Contributions

**Figure 3:**
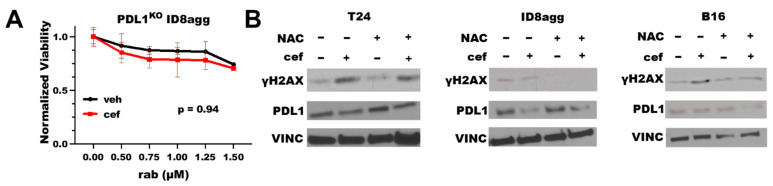
**Cefepime-induced DNA damage and sensitivity to DNA-damaging agents is tumor PDL1-dependent with ROS contributions in distinct lines.** (**A**) MTT viability in PDL1^KO^ ID8agg cells treated with 80 μM cefepime (cef) or DMSO (veh) in combination with indicated concentrations of rabusertib (rab). *p* value by two-way ANOVA. (**B**) Immunoblots of T24, ID8agg, and B16 cells treated with 80 μM cefepime and/or 0.5 mM N-acetyl-L-cysteine (NAC) for γH2AX, PDL1, and loading control vinculin (VINC) (48 h incubation).

To define the PDL1 dependence of the cefepime-mediated improvement in the DNA-damaging-agent efficacy, we tested cefepime combined with rabusertib in genetic PDL1 knock-out (PDL1^KO^) ID8agg cells. Cefepime failed to improve the rabusertib efficacy in PDL1^KO^ ID8agg cells (Figure 3A), which is consistent with the tumor PDL1 dependence of the cefepime efficacy. Some β-lactam antibiotics can induce reactive oxygen species (ROS), which damage DNA [20]. To test the ROS contributions to PDL1-dependent cefepime-mediated DNA damage, we added the ROS scavenger N-acetyl-L-cysteine (NAC) to cultures with cefepime, and we assessed γH2AX as a measure of the DNA damage. In human T24 cells, the γH2AX induction by cefepime was unaltered by NAC (Figure 3B), which implies that the cefepime-mediated DNA damage is not from ROS contributions in this cell line. However, in murine B16 and ID8agg cells, we found that NAC moderately attenuated the γH2AX induction (Figure 3B), which is consistent with a minor role for ROS generation in PDL1 depletion-mediated DNA damage induction by cefepime in distinct tumor models (B16 quantification in Supplementary Figure S2).

### 2.4. Cefepime Improves Rabusertib Sensitivity In Vivo and Skews towards TH1-Polarized Immunity

**Figure 4:**
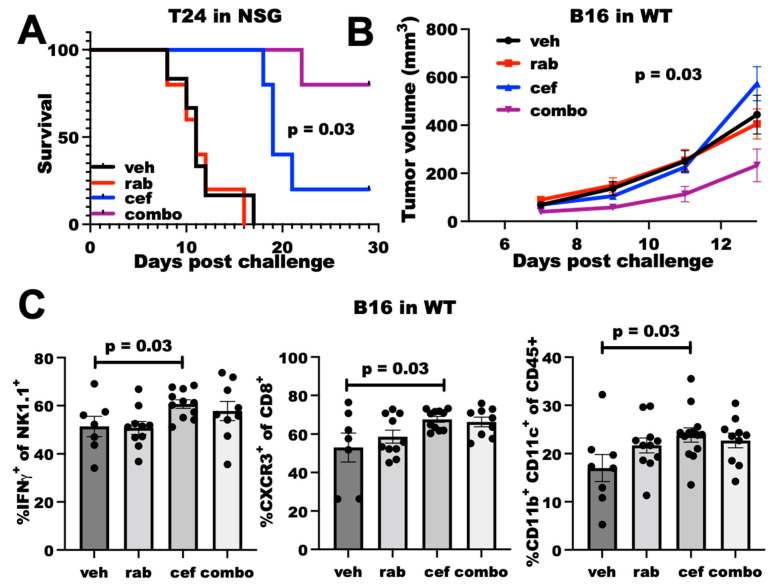
**Cefepime elicits PDL1-dependent rabusertib sensitivity in vivo and skews towards TH1.** (**A**) Survival of NSG mice (*n* = 5 per group) challenged with T24 cells. Mice were treated beginning Day 7 with vehicle (veh), 200 mg/kg cefepime (cef), and/or 2.5 mg/kg rabusertib (rab) daily. *p* value by log-rank test. (**B**) Tumor growth in WT mice challenged with B16 cells treated with vehicle, 5 mg/kg rabusertib daily, and/or 200 mg/kg cefepime twice daily beginning Day 3 post-challenge. *p* value by two-way ANOVA. (**C**) Flow cytometry analyses of immune populations in tumors derived from vehicle-, rabusertib-, cefepime-, and combination (combo)-treated mice. *p* values by unpaired *t* test.

We next tested the *in vivo* efficacy of cefepime at improving DNA-damaging agent efficacy. As cefepime most potently improved Chk1i efficacy *in vitro*, we tested the Chk1i rabusertib in *in vivo* studies. In NSG mice challenged with T24 cells, rabusertib alone was ineffective at improving mouse survival, but cefepime significantly improved mouse survival (Figure 4A), which is similar to genetic PDL1^KO^ T24 [9]. Strikingly, the combination of cefepime with rabusertib potently improved mouse survival over either single agent (Figure 4A), which demonstrates the *in vivo* utility of our *in vitro* findings. These data from severely immunodeficient NSG mice essentially eliminate the microbiota contributions to cefepime antitumor efficacy, as microbes contribute through immune mechanisms [21], which is further supported by the *in vitro* efficacy where no immune mediators are present. Thus, cefepime can improve Chk1i efficacy *in vivo* in an immune-independent manner, which is likely related to augmented DNA damage.

We also tested cefepime efficacy in wild-type (WT) mice, as detrimental immune consequences from antibiotic treatments are a possibility as has been noted for various cancer treatments [22]. In WT mice challenged with B16 cells, neither cefepime nor rabusertib alone controlled tumor growth. However, the combination was significantly effective (Figure 4B). Flow cytometry analysis of tumor-infiltrating cells demonstrated that cefepime increased dendritic cells, CXCR3^+^ CD8^+^ T cells (consistent with TH1-polarized immunity [23]), and IFNγ^+^ NK cells, whereas rabusertib alone did not (Figure 4C). Surprisingly, other immune cell populations and effector cell functions that are typically altered through tumor PDL1 depletion (e.g., IFNγ^+^ CD8^+^ T cells, Granzyme expression) were unaffected by cefepime treatment (Supplementary Figure S3), which suggests differential immune consequences of PDDs. As the immune effects of the combination treatment were similar to cefepime alone, we were unable to ascribe a specific immune mechanism to the *in vivo* treatment efficacy from these data.

### 2.5. PDDs Promote Tumor STING Activation

**Figure 5:**
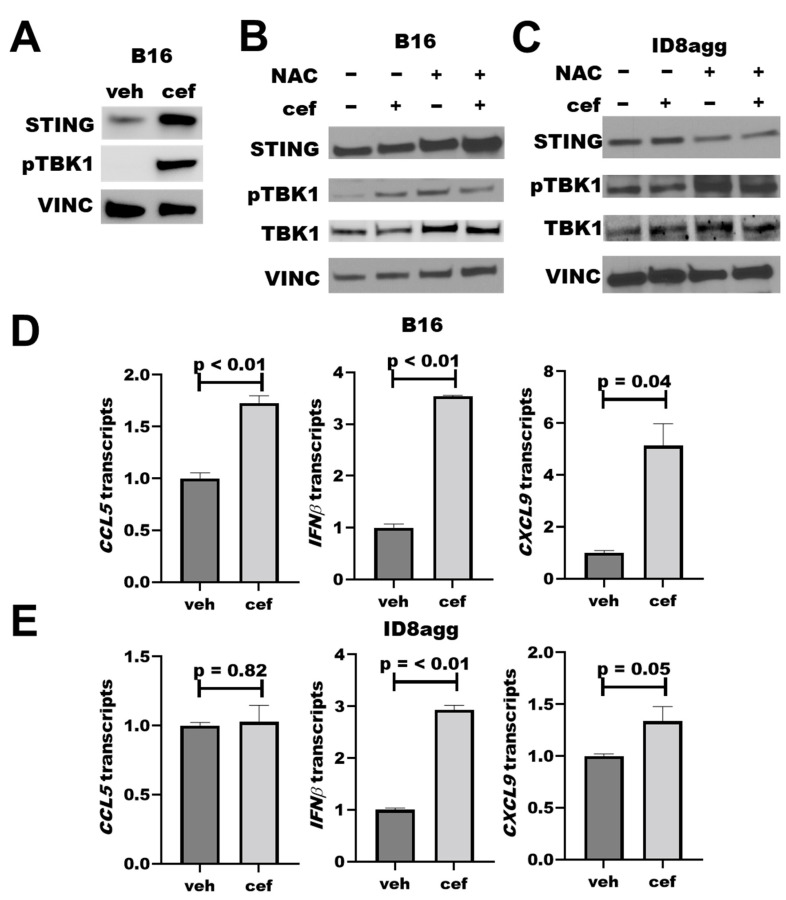
**Cefepime promotes tumor STING activation.** (**A**) Immunoblot for STING, phospho-TBK1 (pTBK1), and loading control vinculin (VINC) of B16 cells treated with DMSO or 80 μM cefepime (cef) for 24 h. (**B**) Western blot for STING, pTBK1, total TBK1, and loading control vinculin in B16 cells treated with 80 μM cefepime and/or 0.5 mM N-acetyl-L-cysteine (NAC) for 48 h. (**C**) Immunoblot for targets as in (**B**) in ID8agg cells treated with 80 μM cefepime and/or 0.5 mM N-acetyl-L-cysteine for 48 h. (**D**) RT-qPCR assessment of normalized gene expression in B16 cells treated as in (**B**). *p* values by unpaired *t* test. (**E**) RT-qPCR assessment of normalized gene expression in ID8agg cells treated as in (**C**). *p* values by unpaired *t* test.

As faulty DNA damage repair can activate the immunogenic STING pathway [24,25], we studied cefepime effects on the STING pathway. Cefepime increased total tumor STING after 24 h of treatment, which could augment STING signals. We observed a significant increase in pTBK1 by immunoblots (Figure 5A), which is a clear marker for functional STING activation and consistent with elevated STING [24]. As β-lactams can induce DNA-damaging ROS [20], which could potentially induce STING signals, we assessed the ROS contributions to the cefepime-mediated STING signals by immunoblot. By using samples as in Figure 3B, which had significant tumor PDL1 depletion with the cefepime treatment, we found that 48 h of cefepime treatment moderately induced pTBK1 with total STING protein unchanged (Figure 5B,C). Although the total STING protein content is unchanged at 48 h versus 24 h of cefepime treatment, elevated pTBK1 signals are indicative of active STING signals. We hypothesize that STING protein content recovers by 48 h of treatment versus elevation at 24 h, although the signals remain active as is evident by elevated pTBK1. Notably, NAC treatment slightly attenuated STING signals in B16 and ID8agg (Figure 5B,C; B16 quantifications in Supplementary Figure S2). These findings support a minor role of ROS in cefepime-induced STING induction. In further support of STING activation, we used RT-qPCR to show increases in the transcription of the downstream STING-activation products *Cxcl9*, *Cxcl10*, *Ccl5*, and *IFNβ* (Figure 5D,E). CXCR3 is the receptor for CXCL9 and CXCL10, and the increase in *CXCL9* transcription supports the *in vivo* immune consequences in Figure 4; however, further investigations are needed for a mechanistic confirmation of cefepime-activated STING in *in vivo* treatment efficacy, as we hypothesize, and for STING effects in PDL1-deficient tumors.

### 2.6. Cefepime Regulates Tumor PDL1 Post-Translationally

**Figure 6:**
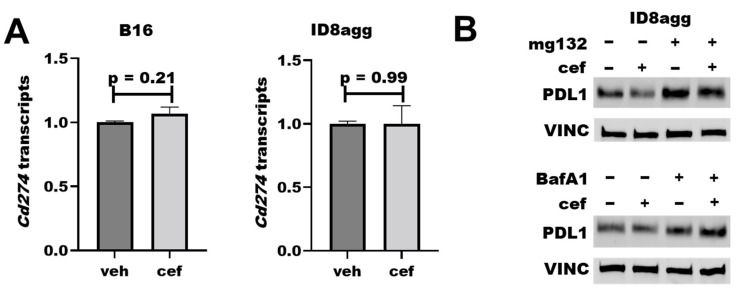
**Cefepime regulates tumor PDL1 post-translationally.** (**A**) RT-qPCR assessment of normalized *Cd274* gene expression of B16 and ID8agg cells treated with DMSO (veh) or 80 μM cefepime (cef) for 48 and 24 h, respectively. (**B**) Western blot of ID8agg cells treated with 0.2 μM mg132 for final 6 h, 100 nM bafilomycin A1 (BafA1) for final 6 h, and/or 80 μM cefepime for 48 h for PDL1 and loading control vinculin (VINC).

To understand how cefepime regulates tumor PDL1 protein content, we first assessed *Cd274* mRNA-encoding PDL1 in ID8agg and B16 cells; however, it was not reduced in either cell line (Figure 6A). We next interrogated protein stability mechanisms through the proteasome inhibitor mg132 and the lysosomal inhibitor bafilomycin A1 in cells collected from the same experiment, which proved the consistency of the tumor PDL1 depletion. Strikingly, mg132 significantly preserved the PDL1 protein content in the presence of cefepime (Figure 6B; quantified in Supplementary Figure S2), which is consistent with ubiquitin-mediated degradation as a cefepime PDL1-depletion mechanism, and which is supported by the increased PDL1 with bafilomycin A1. Additional work is required in order to elucidate the PDL1-depletion mechanism fully.

### 2.7. The Cefepime β-Lactam Ring Appears Dispensable for PDD and Cytotoxic Effects

**Figure 7:**
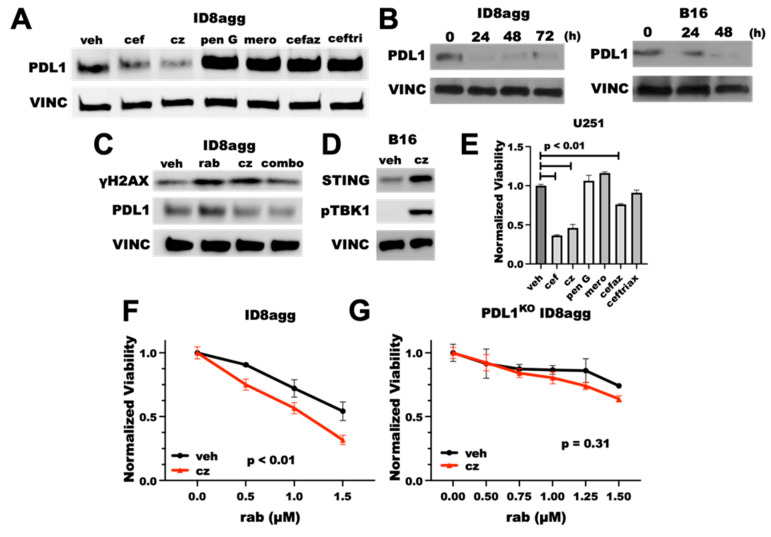
**Tumor PDL1depletion effects of cefepime are likely independent of the β-lactam ring.** (**A**) Immunoblot of PDL1 and loading control vinculin (VINC) of ID8agg cells treated with DMSO (veh) or 80 μM of β-lactam antibiotics cefepime (cef), ceftazidime (cz), penicillin G (pen G), cefazolin (cefaz), ceftriaxone (ceftri), and the carbapenem meropenem (mero) for 48 h. Drugs were replenished daily. (**B**) Immunoblot for PDL1 and loading control vinculin of ID8agg and B16 cells treated with ceftazidime for indicated time points, replenished daily. (**C**) Western blot for γH2AX, PDL1, and loading control vinculin of ID8agg treated with DMSO, 2.5 μM rabusertib (rab), 80 μM ceftazidime, or combination for 72 h. (**D**) Immunoblot of B16 cells treated with DMSO or ceftazidime for 24 h for STING, phospho-TBK1, and loading control vinculin. (**E**) MTT viability of U251 cells treated with 80 μM of β-lactam antibiotics cefepime, ceftazidime, penicillin G, cefazolin, ceftriaxone, and the carbapenem meropenem for 96 h. *p* values by unpaired *t* test. (**F**) MTT viability assay of ID8agg cells treated with rabusertib (rab) with DMSO or 80 μM ceftazidime for 96 h. *p* value by two-way ANOVA. (**G**) MTT viability of PDL1^KO^ ID8agg cells treated with rabusertib, DMSO, or 80 μM ceftazidime for 96 h. *p* value by two-way ANOVA.

Cephalosporins are a class of β-lactam antibiotics, which are reported to have negative effects on treatment outcomes in cancer patients [22]. Thus, we investigated whether the cefepime β-lactam ring is required for its PDD effects. We tested several β-lactam antibiotics for tumor PDL1 depletion, including penicillin G and other β-lactams in the cephalosporin and related antibiotic classes, such as the first-generation cephalosporin cefazolin, the carbapenem meropenem, the third-generation cephalosporin ceftriaxone, and the fourth-generation cephalosporin ceftazidime. Surprisingly, penicillin G, meropenem, cefazolin, and ceftriaxone all induced tumor-PDL1 expression (Figure 7A), which was potentially from DNA damage induction through ROS production. However, ceftazidime potently depleted the tumor PDL1 with a similar efficacy to cefepime (Figure 7A), which we replicated in B16 and ID8agg cells over time (Figure 7B). Ceftazidime suppressed the PDL1 induction in ID8agg cells with rabusertib treatment and induced γH2AX, which phenocopies Figure 2A. cefepime effects (Figure 7C). We next tested STING activation and cytotoxicity, and we found that ceftazidime both induces STING signals through increased STING protein and pTBK1 induction and reduces cell viability similar to cefepime (Figure 7D,E). Most of the other β-lactam antibiotics that were used in our preliminary structure–activity-relationship studies that did not deplete tumor PDL1 also failed to reduce the U251 GBM cell viability, except for modest cefazolin activity (Figure 7E), which merits further investigation. Because ceftazidime is similarly able to induce γH2AX as for cefepime, we tested whether ceftazidime could elicit sensitivity to DNA-damaging agents. Ceftazidime induced rabusertib sensitivity in ID8agg cells similarly to cefepime which was eliminated in PDL1^KO^ cells, implying tumor PDL1 dependence of rabusertib sensitivity (Figure 7F,G). Ceftazidime is structurally very similar to cefepime, whereas the other β-lactam antibiotics that were tested shared few structural similarities, apart from their β-lactam rings (Supplementary Figure S4). These data support the concept that the β-lactam ring is dispensable for PDD effects, and they provide opportunities for structure–activity-relationship studies to define potentially better molecules that mediate tumor PDL1 depletion and that are derived from cefepime/ceftazidime structures.

### 2.8. PDDs Phenocopy Other Genetic Tumor-PDL1^KO^ Outcomes

**Figure 8:**
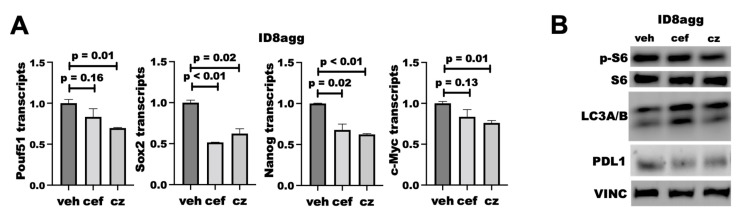
**PDDs phenocopy additional genetic tumor-PDL1-depletion effects.** (**A**) RT-qPCR for normalized stemness-associated gene expression (*Pouf51*, *Sox2*, *Nanog*, *c-Myc)* of ID8agg cells treated with DMSO (veh) or 80 μM cefepime (cef) for 48 h, replenished daily. *p* values by way of unpaired *t* test. (**B**) Immunoblot for phospho-S6, total S6, LC3A/B, PDL1, and loading control vinculin (VINC) in ID8agg cells cultured and replenished daily with 80 μM cefepime (cef) or ceftazidime (cz) daily for two days.

We reported that tumor PDL1 promotes mTORC1 and stemness and suppresses autophagy [7,8,13], all of which are actionable treatment targets, so we tested whether PDDs replicated genetic tumor PDL1 depletion effects on these pathways. We found that cefepime and ceftazidime reduced the transcription of several stemness genes, including *Sox2* and *Nanog* (Figure 8A), which is similar to what we reported for PDL1^lo^ ID8agg clones [13]. We found that both cefepime and ceftazidime induce autophagy through LC3A/B induction in ID8agg cells, which is consistent with our findings on genetic PDL1 depletion [7]. However, they minimally affect mTORC1 activity, seen through phospho-S6, when tumor PDL1 is depleted (Figure 8B; quantified in Supplementary Figure S2). Therefore, cefepime and ceftazidime can phenocopy other tumor-intrinsic PDL1 depletion effects aside from DNA damage and sensitivity to DNA-damaging agents, which could be clinically exploitable.

## 3. Discussion

Although the paradigm of surface-expressed PDL1 engaging immune cell PD1 to thwart antitumor immunity is valid and has led to important ICB drugs, it is incomplete. Recent work demonstrates the immunopathologic import of tumor-intrinsic PDL1 signals, which notably includes their role in the tumor treatment resistance to distinct classes of therapies, including cytotoxic agents, targeted small molecules, irradiation, and immunotherapies (reviewed in [6]). Although genetic tumor PDL1 depletion can define mechanistic insights, it is not a logistically tractable approach to cancer treatment with current technology. Recent interesting reports from several groups have shown that small molecules can deplete tumor-intrinsic PDL1 to alter cancer treatments. For example, FDA-approved verteporfin, which is a benzoporphyrin molecule that is used to treat retinal diseases, depletes PDL1 from distinct human tumor cells and improves PARP inhibitor (PARPi) treatment *in vivo* in mouse ovarian tumors [26]. Curcumin is a natural product that can reduce tumor cell PDL1 content by promoting its ubiquitination and can improve *in vivo* anti-CTLA-4 immunotherapy efficacy in murine 4T1 triple-negative breast cancer [27]. Small molecule inhibitors of PDL1 and PD1 are also described [28,29], but these were generally designed to replace the antibody action of inhibiting PDL1/PD1 interactions at cell surfaces and not to reduce tumor-intrinsic PDL1.

We used a drug screen to identify a variety of FDA-approved drugs that can be repurposed to deplete tumor PDL1 and phenocopy important genetic PDL1 depletion outcomes, including suppressing mTORC1 and tumor stemness, and promoting tumor autophagy. These are actionable pathways that merit additional study and that are not fully addressed here. We found that the fourth-generation cephalosporin cefepime potently reduces tumor PDL1 in a wide variety of human and mouse tumor cell lines to phenocopy the recently described role of PDL1 in DNA damage repair [14,30]. Although the drug screen used up to 10 μM of the candidate drugs to assess the PDL1 with minimal cytotoxic effects, we found that drug concentrations higher than used in the drug screen, and yet still clinically achievable, could inhibit tumor cell growth *in vitro* and *in vivo*.

Cefepime PDL1-dependently increased tumor DNA damage and sensitivity to DNA-damaging agents, including the cytotoxic chemotherapy drug gemcitabine and two small molecule Chk1 inhibitors. We recently reported that tumor PDL1 specifically promotes homologous recombination DNA repair, and its genetic depletion augmented PARPi cytotoxicity *in vitro* and *in vivo* [14]. Cefepime phenocopied genetic PDL1 depletion in augmenting DNA damage and sensitivity to targeted small molecule DNA-damaging agents *in vitro*, but the effects were modest versus genetic PDL1 depletion, and it did not sensitize to PARPi as did genetic PDL1 depletion [14]. This difference in the effect size could be due to tumor-specific factors, incomplete cefepime effects on PDL1-regulated DNA damage repair molecules, incomplete PDL1 depletion, or a requirement for immune contributions, which could be seen *in vivo*. Further work to determine PDD impairment of homologous recombination, sensitivity to PARPi, and whether PDL1-dependent STING activation contributes to *in vivo* efficacy is required.

By contrast, cefepime robustly increased tumor cell death mediated by the cytotoxic chemotherapy drugs gemcitabine and temozolomide, which have mechanisms of cytotoxicity that are distinct from each other [31,32] and that are distinct from small molecule Chk1 inhibitors. These data suggest that cefepime affects DNA damage repair pathways aside from homologous recombination and that it could affect the mechanisms that are specific to the actions of temozolomide and gemcitabine. Much additional work is required in order to understand how cefepime (and ceftazidime) influences cytotoxicity that is mediated by distinct molecules with differing mechanisms of action; however, we hypothesize that the influence on the distinct DNA damage sensing or repair pathways is among the mechanisms, including the specific effects on the Chk2 DNA damage sensing pathway as we recently reported [33]. It is now recognized that tumor PDL1 affects gene product expression through distinct mechanisms, including through gene expression [12,13] and post-translational mechanisms. Tumor PDL1 also promotes sister chromatid cohesion [34] and genomic stability [35], which could contribute to cytotoxic effects in PDL1 depletion. We further found that ROS could contribute to cytotoxicity or DNA damage in selected cell lines. All these mechanistic details require additional work.

*In vivo* data show that cefepime affects immune cells in a distinct manner, including by potentially augmenting Th1 polarization, and shows signs of STING activation, such as an increase in CXCR3^+^ immune cells [23,25]. It will be important to understand how the cefepime-mediated immune effects are generated, as well as their contributions to the cefepime *in vivo* efficacy. We predict that increased tumor STING activation with cefepime will contribute to and could improve ICB efficacy, despite the reduced tumor PDL1 expression. In support, we just reported that the PDD chlorambucil renders αPDL1-resistant tumors αPDL1-responsive [36]. These lines of investigation are also important, as many antibiotics appear to worsen cancer treatments including ICB [22]. Many cytotoxic agents also increase tumor immunogenicity, including through immunogenic cell death [37], warranting further investigation.

The effects of cefepime and rabusertib on several aggressive cell lines, particularly GBM, are notable for their potency. PDDs can thus help to fill the GBM treatment gaps, as most highly express PDL1 [38]. Notably, both cefepime (and ceftazidime) and rabusertib cross the blood–brain barrier, which is required for effective systemic GBM treatment. GMB has demonstrated a poor response to single-agent ICB, and combination ICB is currently in clinical trial; thus, combining PDDs with single agent ICB or DNA damage-inducing molecules could improve GBM treatments and outcomes, as we have shown with the PDD chlorambucil in ovarian cancer [36].

Our work has limitations in addition to those mentioned above. We have yet to show that *in vivo* cefepime efficacy is tumor PDL1-dependent, which we expect, as well as whether it is tumor-selective in PDL1 depletion, both of which we demonstrated for the PDD chlorambucil [36]. Some PDL1 effects are related to the subcellular location, such as the cytoplasmic PDL1 regulation of DNA damage repair gene expression [30] and the nuclear PDL1 regulation of αPD1 resistance and pyroptosis [12,13]. We have yet to understand if the cefepime effects on sensitization to DNA-damaging agents is related to a specific subcellular PDL1 location and whether specific PDDs deplete PDL1 from specific subcellular locations. Furthermore, the precise mechanisms for the sensitization to these agents has yet to be defined, as do the immune contributions. Finally, we expect the PDD effects to differ by tumor type, which we demonstrated here; however, much work is needed in order to understand these differences, which likely relate to the underlying tumor mutational landscape among other considerations.

In summary, we show that pharmacologic tumor PDL1 depletion increases DNA damage, sensitizes a wide variety of human and mouse tumor cells to cytotoxicity from distinct DNA-damaging agents, augments the treatment efficacy to a Chk1 inhibitor *in vivo*, and activates STING signals, all of which are clinically translatable. Cefepime was our proof-of-concept FDA-approved agent as a PDD, but it has limitations. Although it is inexpensive, relatively safe, and well tolerated, its antimicrobial effects (which likely do not contribute to its treatment efficacy here) and its short half-life could be problematic. Our initial structure–activity-relationship studies suggest that the antimicrobial β-lactam ring is not required for the outcomes that we observed, nor are its antimicrobial effects likely mechanistic in the treatment efficacy here. Better agents could be designed from the structures that have been identified here or from among other PDDs that we are now evaluating. Combination therapies of PDDs with other immunotherapies are also worth exploring. Pharmacologic tumor PDL1 depletion merits additional studies for its clinical ability to target other PDL1 effects, such as mTORC1 signals and rapamycin sensitivity, and for its potential to improve selected ICB as we have demonstrated with chlorambucil.

## 4. Materials and Methods

**Cell lines and constructs.** B16 murine melanoma cells were obtained from ATCC. ID8agg cells were generated through serial *in vivo* passage, as we previously described [8]. T24 human bladder cancer cells were a gift from Dr. Robert Svatek, MD. Cell lines were not revalidated for this study, except for T24. B16 and ID8agg cells were cultured in RPMI-1640 medium, while T24 was cultured in McCoy’s medium. GL261 murine glioblastoma cells were a kind gift from Sandeep Burma, PhD. GL261 were cultured in DMEM. U251 human glioblastoma cells were a gift from the Agenus Corporation (Lexington, MA, USA) and were cultured in EMEM. PDL1^KO^ cell lines were generated by using commercially available CRISPR/Cas9 plasmids, as we previously described, and were validated through flow cytometry, Western blot, and sequencing [9].

**Chemicals, reagents, and X-rays.** Gemcitabine was purchased from Sigma-Aldrich (St. Louis, MO, USA) and was diluted in sterile water for *in vitro* studies. Cefepime was purchased from Sigma-Aldrich and was diluted in DMSO for *in vitro* studies, while *in vivo* USP cefepime was obtained from Oakdell Pharmacy (San Antonio, TX, USA) and was diluted in sterile 0.9% NaCl (Intermountain, West Joran, UT, USA). Penicillin G, meropenem, cefazolin, and ceftriaxone were also obtained from Oakdell Pharmacy and were used without further manipulations. Temozolomide was obtained from the Mays Cancer Center (San Antonio, TX, USA) and was diluted in DMSO. N-acetyl-L-cysteine (NAC) was purchased from Sigma-Aldrich. Gemcitabine was purchased from Sigma-Aldrich. Rabusertib was purchased from Selleck Chemicals (Houston, TX, USA) and was diluted in DMSO for in vitro studies. For in vivo studies, the rabusertib vehicle was 5% DMSO, 30% PEG300 (Selleck Chemicals), 5% Tween 80 (Sigma-Aldrich), 5% Propylene Glycol (Sigma-Aldrich), and sterile 1× PBS.

***In vitro* proliferation and viability.** For MTT cell viability assays, 1000 cells/well were plated in 96-well plates in appropriate growth medium. Cells were treated with indicated agents the following day and were incubated for up to 96 h. The drug concentrations shown in these data were carefully optimized in preliminary experiments. A total of 20 microliters of 5 mg/mL 3-(4,5-dimethylthiazol-2-yl)-2,5-diphenyl tetrazolium bromide (MPBio, Solon, OH, USA) was added/well, and then the plates were incubated for 2 h at 37 °C. Medium was then decanted, and wells were resuspended in 200 microliters of DMSO. Absorbance was read at 490 nm.

**RT-qPCR.** Cells were lysed with TRIzol (Invitrogen, Waltham, MA, USA) and total RNA was isolated, according to the manufacturer’s recommendations. A NanoDrop 1000 was used to determine the quantity and quality of the RNA. cDNA was reverse transcribed from RNA with SuperScript III Reverse Transcriptase (Invitrogen). Gene expression was determined by quantitative real-time RT-PCR (qRT-PCR) by using TaqMan Gene Expression Assays (Applied Biosystems, Waltham, MA, USA) and a QuantStudio 3 (Applied Biosystems). Values were normalized to GAPDH, which was used as a housekeeping gene with the Applied Biosystems Data Analysis software by using the ΔΔCq method. All TaqMan primers were purchased from ThermoFisher Scientific (Waltham, MA, USA) and were validated by the manufacturer.

**Immunoblots and antibodies.** Cells were lysed with 1× TN1 lysis buffer (125 mM NaCl, 50 mM Tris, 10 mM EDTA, 1% Triton X-100, 10 mM Na_4_PO_7_, 10 mM NaF) with a 1:100 Halt protease/phosphatase inhibitor cocktail (ThermoFisher Scientific). Protein concentrations were measured by BCA Assay (ThermoFisher Scientific). A total of 40 micrograms of protein per sample were run on 4 to 15% DS-PAGE Precast TGX gels (Bio-Rad, Hercules, CA, USA) and were transferred to 0.2-micron nitrocellulose membranes (Bio-Rad) by using a Trans-Blot Turbo transfer system (Bio-rad). Membranes were incubated with the appropriate primary antibodies overnight at 4 °C, washed with 1× TBST, and incubated in species-matched horseradish peroxidase-conjugated secondary antibodies for 2 h at ambient temperature. After wash with 1× TBST, membranes were incubated with Western Lightening Plus reagent (PerkinElmer, Austin, TX, USA) or SuperSignal West Pico PLUS (ThermoFisher Scientific) for chemiluminescent detection. Antibodies purchased from Cell Signaling Technology (Danvers, MA, USA) include phospho-histone H2AX (#9718), PDL1 (#13684), vinculin (#13901), GAPDH (#2118), p-TBK1 Ser172 (#5483), TBK1 (#3013), STING (#13647), LC3 A/B (#12741), S6 ribosomal protein (#64108), and phospho-S6 ribosomal protein (#4858). Mouse-reactive PDL1 antibody was purchased from Abcam (Cambridge, UK) (ab213480). The incubation times used for the PDD experiments differed among distinct cell lines on the basis of the differential optimal time to maximal PDL1 depletion in each cell line.

**Mice.** Wild-type C57BL/6J (BL6) and NOD.Cg-Prkdcˢᶜ^i^ᵈ Il2rgᵗᵐ¹ᵂʲˡ/SzJ (NSG) mice were originally purchased from Jackson Labs (Bar Harbor, ME, USA) then bred in-house and maintained under pathogen-free conditions. All animal studies were approved by the UT Health San Antonio Institutional Animal Care and Use Committee.

***In vivo* tumor challenges and treatments**. NSG mice were challenged with 2 × 10^6^ T24 cells in 1:1 PBS:Matrigel (Corning, Corning, NY, USA) subcutaneously on each flank (*n* = 2 tumors per mouse). BL6 mice were challenged with 0.5 × 10^6^ B16 cells subcutaneously on each flank in PBS. Rabusertib (5 mg/kg) and vehicle (5% DMSO, 30% PEG300, 5% Tween 80, 5% Propylene Glycol, 55% PBS) were administered daily, beginning as indicated in the text. Cefepime or sterile 0.9% NaCl vehicle were administered at 200 mg/kg/day, beginning as indicated in the text.

**Flow cytometry.** Mice were sacrificed by cervical dislocation after isoflurane anesthesia. Tumors were excised and then strained in 100-micron cell strainers into serum-free RPMI-1640 medium to create single-cell suspensions. Total cell numbers per sample were counted with a Vi-Cell-XR cell counter (Beckman Coulter). A total of 5 × 10^6^ cells per sample were transferred to U-bottom 96-well plates. Dead cells were excluded by the LIVE/DEAD Fixable Blue Dead Cell Stain Kit for UV excitation (ThermoFisher Scientific). Samples were incubated with anti-CD16/32 (Biolegend) for 30 min at 4 °C and were shielded from light to prevent nonspecific binding. Cells were stained with surface antibodies at 1:100 for 30 min at 4 °C while protected from light. Surface antibodies include αPD1 (566515), αCD11b (748700), αCD4 (612952), αCD8 (563786), αCXCR3 (748700), and αB220 (612972) from BD Biosciences (Franklin Lakes, NJ, USA); αCD3 (80-0032-U100), αNK1.1 (20-5941-U100), and αCD11c (60-0114-U100) from TONBO Biosciences (San Diego, CA, USA); αCD45 (58-0451-82) from Invitrogen; and αCD11b (101226) from BioLegend (San Diego, CA, USA). Cells were washed with FACS buffer (1:50 FBS in 1× PBS) and were then activated with Cell Activator Cocktail (Biolegend), which contained phorbol 12-myristate 13-acetate, ionomycin, and brefeldin A in a CR10 medium (RPMI-1640, 10% FBS, L-glutamine, sodium pyruvate, nonessential amino acids, penicillin/streptomycin, HEPES buffer) for 6 h to perform intracellular cytokine staining. Cells were washed and were then fixed and permeabilized by using the Foxp3/Transcription Factor Staining Buffer Kit (TONBO Biosciences). After fixation and permeabilization, the samples were incubated with intracellular antibodies and diluted to 1:100 at 4 °C for 30 min while protected from light. Intracellular antibodies include αFOXP3 (ThermoFisher Scientific, 15-5773-82), αIFNγ (BD Biosciences, 612769), and αGranzyme B (Biolegend, 515408). Data were acquired by using a Cytek Aurora flow cytometer (Cytek Biosciences, Fremont, CA, USA) and were analyzed with FloJo software V.10.7.1 (BD Biosciences).

**Generation of Figure 1A and Supplementary Figure S3.**Figure 1A was generated by using BioRender software. Images of structures of β-lactam antibiotics were adapted from The National Library of Medicine National Center for Biotechnology Information PubChem database of compound summaries. Images are cited as follows: National Center for Biotechnology Information (2022). PubChem Compound Summary for CID 5479537, cefepime; National Center for Biotechnology Information (2022). PubChem Compound Summary for CID 5481173, ceftazidime; National Center for Biotechnology Information (2022). PubChem Compound Summary for CID 5904, penicillin G; National Center for Biotechnology Information (2022). PubChem Compound Summary for CID 441130, meropenem; National Center for Biotechnology Information (2022). PubChem Compound Summary for CID 33255, cefazolin; National Center for Biotechnology Information (2022). PubChem Compound Summary for CID 5479530, ceftriaxone.

**Statistics**. Statistical analyses of all data were performed using PRISM software (version 9.3.1, GraphPad, San Diego, CA, USA), with the significance defined as *p* ≤ 0.05. Data for in vivo experiments are represented as mean ± standard error of the mean, while in vitro data are represented as mean ± standard deviation. Survival significance was determined by log-rank test. Tumor growth and viability curves were analyzed by two-way ANOVA. All other data were analyzed with unpaired *t*-test. Outliers were identified by Grubbs’ test, used only once per data set, and removed from analysis.

## Data Availability

Not applicable.

## Supplementary Materials

The following supporting information can be downloaded at: https://www.mdpi.com/article/10.3390/ijms23095129/s1.
