# Supplementary material for: Pharmacologic Tumor PDL1 Depletion with Cefepime or Ceftazidime Promotes DNA Damage and Sensitivity to DNA-Damaging Agents
Source: Int J Mol Sci. 2022 May 4;23(9):5129. doi: 10.3390/ijms23095129 (PMC9099860; doi:10.3390/ijms23095129)
Supplement: Supplementary file 1 [file ijms-23-05129-s001.zip › ijms-1685931-supplementary.pdf]

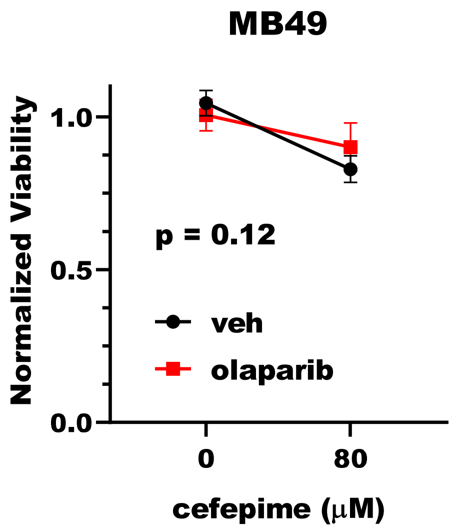

1. Cefepime does not induce PARP inhibitor sensitivity. MTT viability assay of MB49 cells treated with vehicle (DMSO) or 2.5 μM olaparib in combination with 80 μM cefepime for 96 hours. p value by two-way ANOVA.

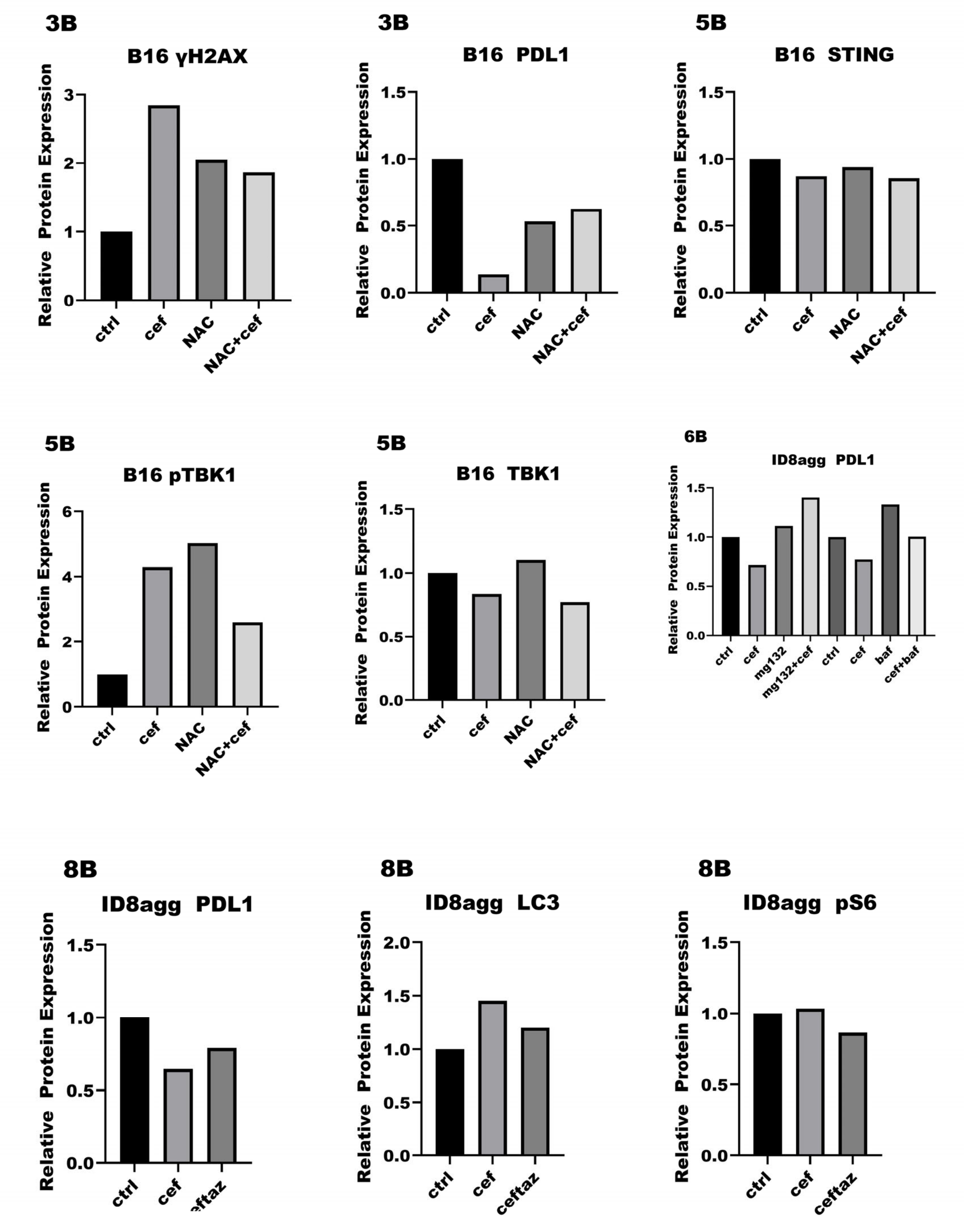

Figure S2. Quantifications of select immunoblot images. Quantifications of immunoblot images of specific targets from indicated figures obtained with ImageJ or Biorad ImageLab software.

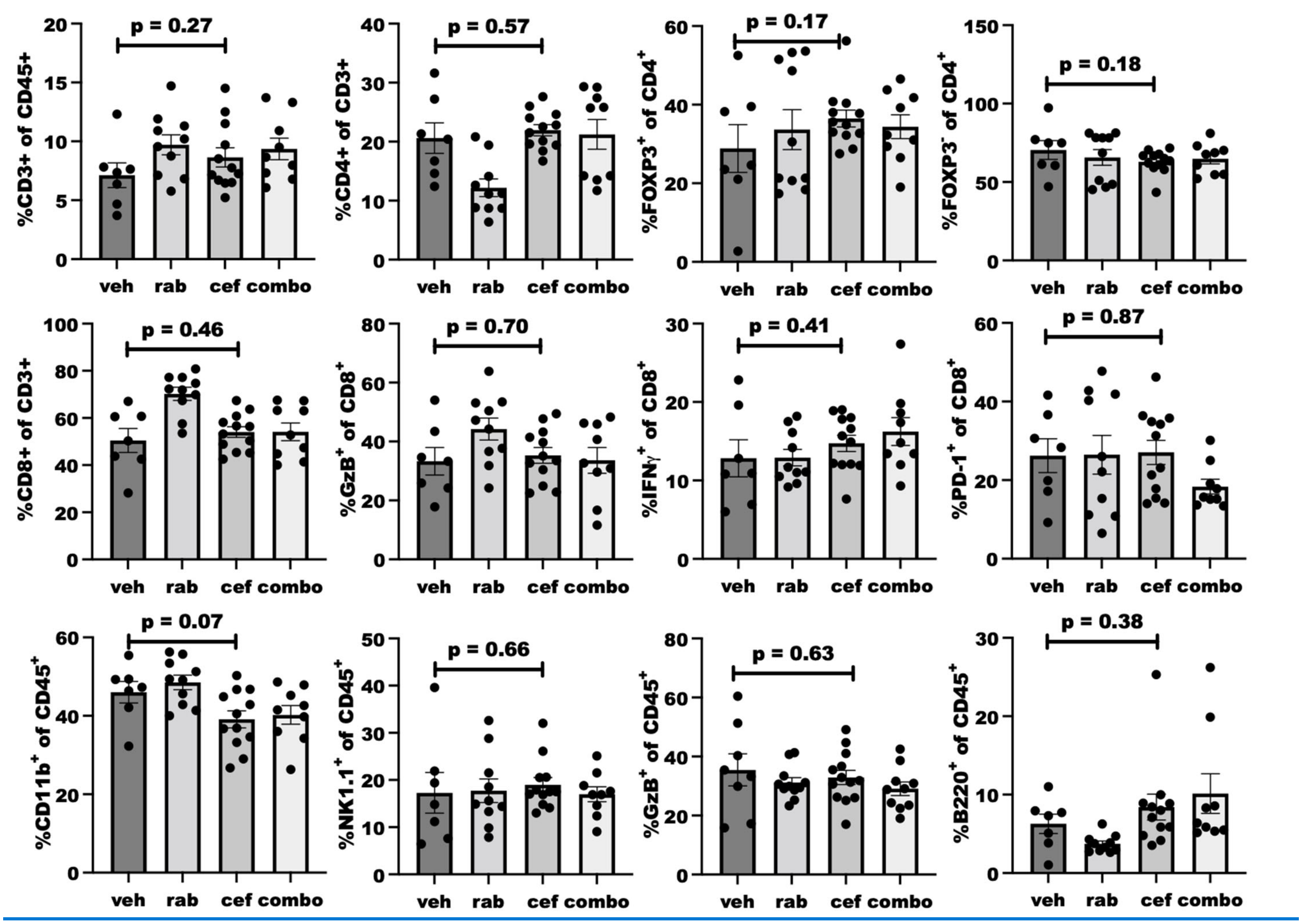

Figure S3. Cefepime treatment effects on immune cell populations *in vivo*. Flow cytometry analysis of immune cell populations within B16 tumors isolated from WT mice treated with vehicle (veh), rabusertib (rab), cefepime (cef), or combination (combo) as in **Fig. 4B**. p values by unpaired t test.

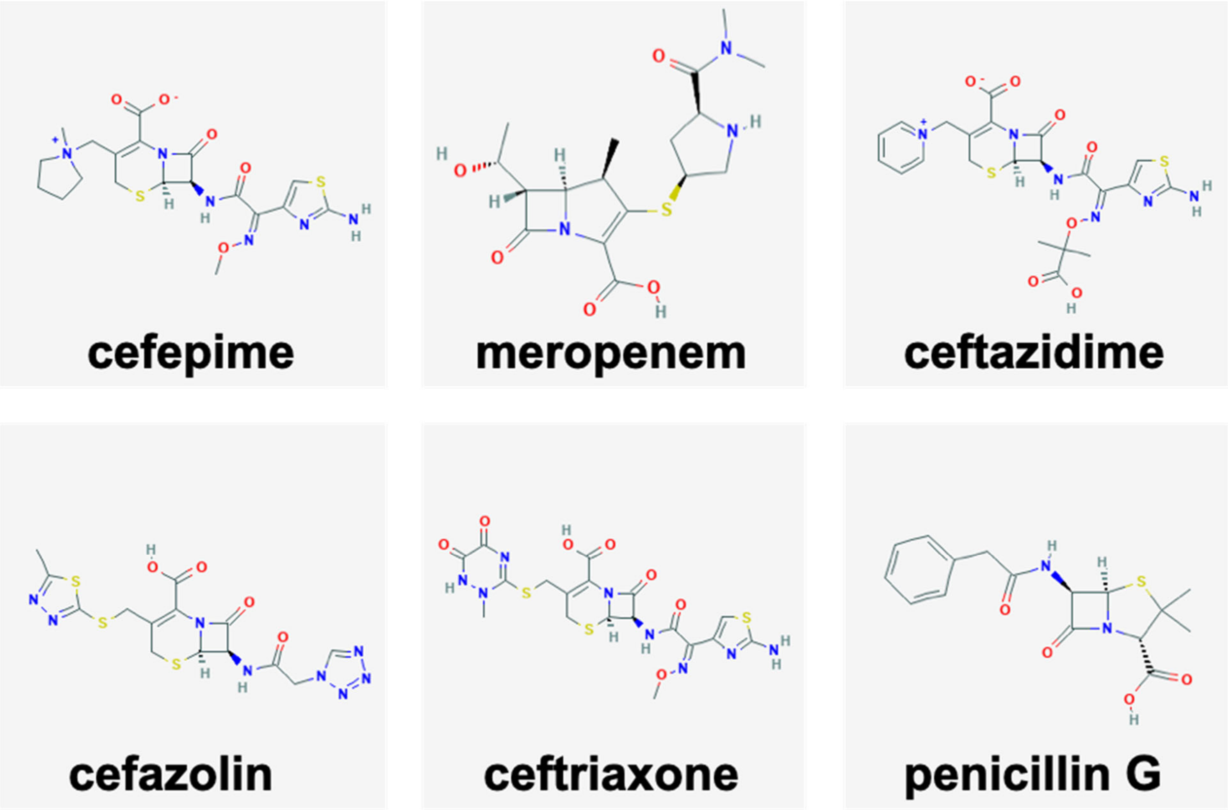

Figure S4. Structures of β-lactam antibiotics for structure activity relationship potential. Compilation of images adapted from the National Library of Medicine – National Center for Biotechnology PubChem database.
